# Very Compact Waveguide Orthomode Transducer in the K-Band for Broadband Communication Satellite Array Antennas

**DOI:** 10.3390/s23020735

**Published:** 2023-01-09

**Authors:** Nelson J. G. Fonseca

**Affiliations:** Antenna and Sub-Millimetre Waves Section, European Space Agency, 2201 AZ Noordwijk, The Netherlands; nelson.fonseca@esa.int

**Keywords:** orthomode transducer, passive waveguide component, dual-polarized phased array antenna, broadband communication satellite

## Abstract

A very compact waveguide orthomode transducer (OMT) is described in this paper. The design is characterized with a twofold rotationally symmetric cross-section in the probing area, adapted from a side-coupling OMT design, simultaneously enabling low port-to-port coupling and high cross-polarization discrimination (XPD) over a fractional bandwidth of about 15–20%. Compared to previously reported compact waveguide OMTs, the proposed design is simpler, thus facilitating its manufacture at millimeter-wave frequencies. The concept is demonstrated with a design in the K-band for a broadband communication satellite downlink over the frequency band of 17.3–20.2 GHz. For test purposes, transitions to standard waveguide WR42 are included, and the OMT is assembled with a conical horn antenna. The measured reflection and coupling coefficients are below −19.5 dB and −22.9 dB, respectively, over the nominal bandwidth, and they are in good agreement with the simulation’s results. The on-axis XPD, measured in an anechoic chamber, is better than 30 dB over the nominal bandwidth, which is also in line with simulations. The proposed waveguide OMT may be designed to fit in a lattice below one wavelength at the highest operating frequency, which is desirable for dual-polarized closely spaced array antennas in low and medium Earth orbit communication satellite systems. The simple mechanical design of the proposed OMT makes it particularly appealing for additive manufacturing techniques, as demonstrated with a variant of the design having folded single-mode waveguides, which preserves the RF properties of the original design.

## 1. Introduction

Waveguide orthomode transducers (OMTs), or dual-mode transducers, are microwave components that enable transmitting two independent electromagnetic signals through the same common waveguide, which is typically a square or circular waveguide. This functionality is essential in communication and radar antenna systems implementing polarization diversity. This waveguide component is widely used in communication satellite antennas to increase the overall system capacity, which is of particular importance in next-generation very high throughput satellite (VHTS) systems [1,2]. OMT design is an important and active field of research, as indicated by the number of recent publications and patents on the topic [3].

One of the first and simplest OMT designs, introduced in the 1950s [4], consists of implementing a side coupling waveguide with a longitudinal slot. Using either blades or a change in cross-section for an adequate design of the in-line transition [5,6,7], only one mode can propagate along the longitudinal direction, resulting in the orthogonal mode being directed to the side-coupling waveguide. This configuration provides naturally very high port-to-port isolation, as orthogonal modes are below the cut-off frequency in respective ports. An alternative implementation of this solution comprises a folded in-line port such that both single-mode ports are opposite to each other, resulting typically in a more compact implementation along the longitudinal direction [8,9,10,11]. However, these configurations are not suited for closely spaced arrays required for future broadband communication satellite systems [12], as the side-coupling port results in a footprint twice that of the waveguide’s broad wall along the coupling direction.

Existing OMT solutions with their footprint fitting within the common waveguide cross-section are typically based on septum designs. These components were first introduced and are still mostly used in waveguide antenna designs to provide dual-circular polarizations [13,14,15,16]. The septum is sized to produce a 90${}^{\circ }$ phase difference between the two orthogonal linearly polarized modes of the common waveguide, resulting in the two single-mode ports producing right-hand and left-hand circular polarization signals. The OMTs described in [17,18] make use instead of a 180${}^{\circ }$ septum design that provides slanted dual-linear modes in the common waveguide with reference to the single-mode waveguide orientation (i.e., $\pm 45^{\circ }$ with reference to the fundamental modes of the common waveguide). The OMT design described in [19] advantageously combines a 90${}^{\circ }$ septum and a 90${}^{\circ }$ iris polarizer to provide flexibility in the orientation of the linearly polarized modes in the common waveguide. This solution also reports a broader frequency bandwidth operation when compared to [17]. However, the designs based on septums are generally quite long, with an in-line dimension that is typically 3 to 4 times the waveguide’s broad wall dimension.

A promising alternative design with a reduced footprint would comprise a folded standard turnstile junction as proposed in [20]. A turnstile waveguide OMT would still lead to a lengthy component because of the combination network required. Two-probe waveguide OMT designs with transversal coupling ports, which may be seen as simplified turnstile junctions, have been reported in [21,22]. Combined with the folding approach described in [20], it could produce the most compact waveguide OMT designs. However, the solutions proposed in [21,22] rely on small features, such as small cylindrical or cross-shaped pins, requiring very high precision milling.

Here, we investigate the adaptation of the compact side-coupling waveguide OMT design with twofold rotational symmetry introduced in [23,24] to a two-probe waveguide OMT with transversal coupling ports. This results in a much simpler OMT design, preserving a compact form factor and the performance of equivalent waveguide OMT solutions. The advantages of the proposed waveguide OMT design are twofold:

- The probing cavity is compact and robust, avoiding small features present in similar previously reported designs [21,22];
- The probing waveguides can be folded [20], providing a design compatible with additive manufacturing constraints when printing along the longitudinal axis of the common waveguide.

These features make the proposed waveguide OMT design particularly suitable for large arrays with an element spacing below a wavelength, which may find applications in next-generation broadband communication satellites.

The paper is organized as follows. Section 2 provides a description of the proposed concept and reports the design, manufacture, and test of an OMT operating in the portion of the K-band allocated to broadband communication satellite’s downlink. Section 3 discusses the adaptation of the design that implements the folding approach introduced in [20] and highlights the benefits in terms of size reduction and suitability of the design for additive manufacturing techniques. Finally, Section 4 provides some conclusions and perspective on future works.

## 2. Very Compact Waveguide OMT

### 2.1. Design Description

When compared to a symmetrical four-probe waveguide OMT, the asymmetric geometry of a two-probe waveguide OMT typically degrades the port-to-port coupling and/or cross-polarization discrimination (XPD), which is defined as the ratio of the co-polarized to cross-polarized electric fields in the common waveguide. This problem was solved in [21] by using a dual-pin matching element and in [22] by using a cross-shaped matching element. However, the small features of these matching elements have an impact on manufacturing lead times and costs when using standard computer numerical control (CNC) milling techniques. They also make them particularly sensitive to manufacturing errors in millimeter-wave frequencies, independently of the manufacturing technique used, which is not desirable for the design of large closely spaced array antennas considered for future broadband communication satellites [12]. There is an interest in simplifying the design of compact dual-probe waveguide OMTs while maintaining simultaneously low port-to-port coupling and high XPD values.

**Figure 1:**
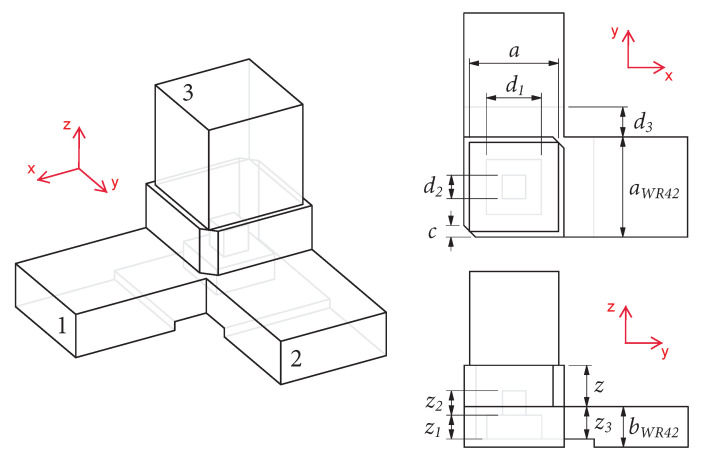
Isometric CAD view of the proposed very compact two-probe waveguide OMT with port numbering (**left**) and associated design parameters in top CAD (**top right**) and side CAD views (**bottom right**).

A similar issue is observed in two-probe waveguide OMTs with longitudinal slots, and it was solved in [23,24] by using a waveguide cross-section with twofold rotational symmetry. This feature is mechanically simple and provides additional degrees of freedom, enabling the fine-tuning of the response of the OMT. This approach is applied here to the design of a very compact dual-probe waveguide OMT in the K-band. A common waveguide with a square cross-section chamfered on two opposite corners provides the twofold rotational symmetry illustrated in Figure 1. As detailed in [24], this twofold rotational symmetry produces two slant fundamental modes in the common waveguide with slightly different propagation characteristics, enabling the introduction of a phase correction between them. This is a well-known feature used in the design of polarizing screens where the phase difference is set to $\pm 90^{\circ }$ or $\pm 270^{\circ }$ to produce circular polarization [25,26,27]. In linearly polarized OMT designs, only a small phase correction is required to compensate for the cross-polarized electric field component produced by the asymmetric junction design. It was found that this feature may also be used within the probing area to control the port-to-port coupling, although the two effects are antagonistic in the OMT configuration discussed in [24], i.e., reducing the cross-polarized electric field component in the common waveguide leads to increased port-to-port coupling while reducing port-to-port coupling results in higher cross-polarized electric field component.

Interestingly, the design reported here is observed to have slightly different operations when compared to the design in [24]. The key difference between two-probe waveguide OMTs having either longitudinal or transversal ports is that the latter requires matching elements in the probing area to achieve low reflection, while the former may be designed without any matching element. The combination here of a simple and symmetric two-step matching element, implemented in the centre of the waveguide OMT and combined with the twofold rotationally symmetric common waveguide cross-section, enables simultaneously controlling the port-to-port coupling and the cross-polarized electric field component. For comparison, the linearly polarized OMT design in [24] having similar characteristics requires two common waveguide portions with different asymmetric cross-sections, one providing port-to-port coupling reduction and the other correcting for the cross-polarized electric field component. This configuration results in a less compact design with about twice the length of the waveguide’s broad wall dimension along the longitudinal direction. Thus, the waveguide OMT design reported here has some clear advantages for single-band microwave systems.

For test purposes, the conical horn design in [24] is reused here, thus constraining the cross-section of the common waveguide at the interface relative to the conical horn (square waveguide of side a=9.5 mm), which is labeled port 3 in Figure 1. The horn comprises a square-to-circular waveguide transition with a cylindrical section of 12.5 mm in diameter and 6.5 mm in length followed by a flare that is 12 mm long, leading to a circular aperture of 25 mm in diameter. The horn was designed to have a reflection coefficient below −30 dB over the band of interest and is, thus, expected to have little impact on the performance of the OMT. The two single-mode ports, labeled port 1 and port 2 in Figure 1, require a transition to the standard waveguide WR42 (10.668 mm × 4.318 mm) for test purposes. This transition is simplified here by setting the side of the probing cross-section equal to the width of the WR42 standard waveguide. All design parameters are defined in Figure 1.

A full-wave model was implemented in Ansys HFSS and optimized over the frequency band of 17.3–20.2 GHz, corresponding to the range allocated to broadband communication satellite’s downlink in the K-band. The design targets include reflection and coupling coefficients below −20 dB and an XPD that is higher than 30 dB over the operating band. The optimized values for all design parameters are reported in Table 1. These values take into account a milling radius of 1.1 mm applied to outer edges in the full-wave model in order to be representative of the manufactured design. The simulated reflection coefficients are better than −23.2 dB over the operating band for port 1 and 2, while the simulated port-to-port coupling coefficient is below −23.3 dB. The XPD is better than 30.3 dB over the nominal band.

### 2.2. Experimental Results

**Figure 2:**
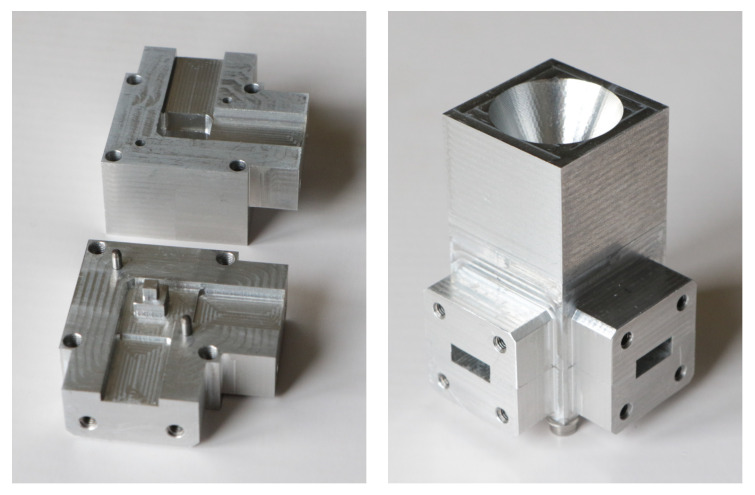
Manufactured very compact dual-probe waveguide OMT prototype: separated parts (**left**) and assembled parts, including the conical horn for test purposes (**right**).

A prototype was manufactured using high-precision milling. The waveguide OMT is composed of two parts, as shown in Figure 2, and assembled with alignment pins for higher precision. The conical horn used for test purposes is also shown in Figure 2. Note that the simple mechanical design of the OMT is compatible with a mill diameter of 2 mm. A much smaller mill diameter is required for the designs in [21,22], leading to higher CNC machining time and thus higher costs, while also resulting in higher sensitivity to manufacturing tolerances.

**Figure 3:**
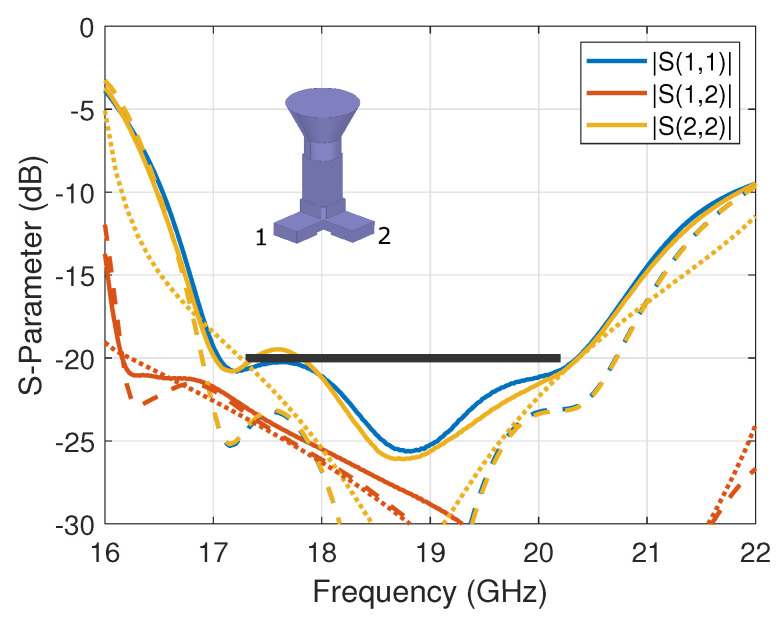
Scattering parameters of the compact dual-mode OMT assembled with a conical horn, comparing measured (*solid lines*) and simulated (*dashed lines*) results. The simulated standalone OMT results are overlaid in *dotted lines*. The specifications are marked with a *black thick line*.

The complete assembly was tested in the compact antenna test range (CATR) at ESA-ESTEC, The Netherlands. The measured scattering parameters (reflection and coupling coefficients) are reported in Figure 3 in *solid lines*, where they are also compared to the simulation’s results in *dashed lines*. A reasonably good agreement was obtained with some small discrepancies, particularly with respect to the reflection coefficients; this may be attributed to manufacturing and assembly (e.g., alignment) errors, which were evaluated to be within ±30 μm. The measured reflection coefficients are better than −19.5 dB, while the measured port-to-port coupling coefficient is better than −22.9 dB over the nominal operating band, corresponding to a fractional bandwidth of 15%. Note that the design is constrained here by the horn cross-section and the standard waveguide width. These parameters may be used as additional optimization variables with the expectation of improving the scattering parameters further, possibly extending the fractional bandwidth up to about 20%. As a reference, the simulated results of the standalone OMT (i.e., without the horn) are reported in Figure 3 in *dotted lines*. As anticipated from the dual-linear operation of the OMT, the horn has very limited impact on port-to-port coupling. The impact on the reflection coefficient is more visible but remains within the target value over the nominal operating band.

**Figure 4:**
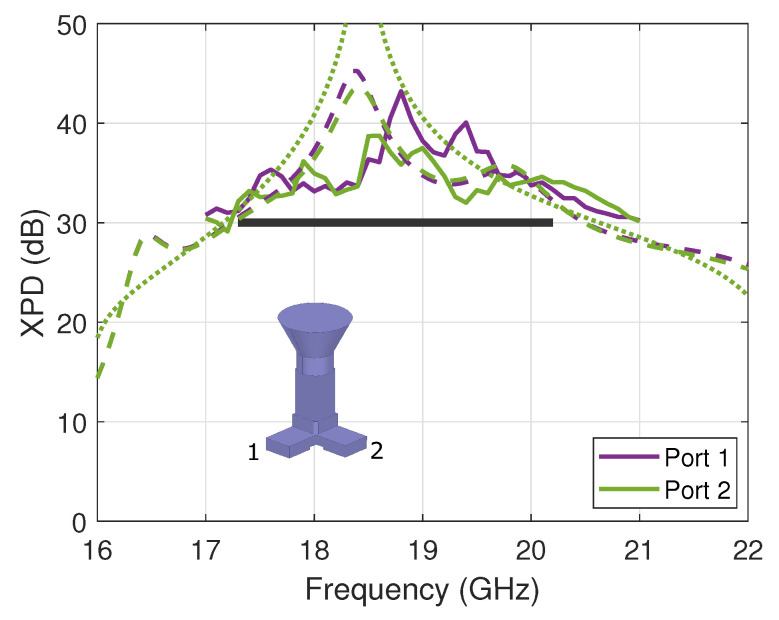
XPD of the compact waveguide OMT assembled with a conical horn (on-axis value), comparing measured (*solid lines*) and simulated (*dashed lines*) results. The simulated standalone OMT results are overlaid in *dotted lines*. The specifications are marked with a *black thick line*.

The experimental evaluation of the XPD of the OMT was performed by measuring the on-axis co- and cross-polarized electric field components in a far-field setup, taking advantage of the symmetry of the horn’s design. However, the measurement results proved to be sensitive to room scattering and probe imbalance, specifically the lower levels of cross-polarized field. A dual-polarized probe is used in the CATR with some residual error between the horizontal and vertical components. These errors are filtered out by averaging two probe orientations with angular values $\phi =0^{\circ }$ and $\phi =90^{\circ }$. The corresponding results are reported for both ports in Figure 4 in *solid lines* and compared to the full-wave simulation’s results in *dashed lines*. The measured XPD of both ports is better than 31.2 dB over the frequency band of interest. The discrepancies between the performance of port 1 and port 2 are not only attributed to manufacturing errors but also measurement uncertainties. Overall, the results are in line with predictions and validate the proposed concept of the two-probe waveguide OMT. Here, the performance of the standalone OMT is also reported in Figure 4 in *dotted lines* as reference, confirming the very little impact of the horn design. Next, we demonstrate that the proposed design maintains similar RF properties when introducing folded single-mode ports, as proposed in [20].

## 3. Very Compact Waveguide OMT with Folded Ports

**Figure 5:**
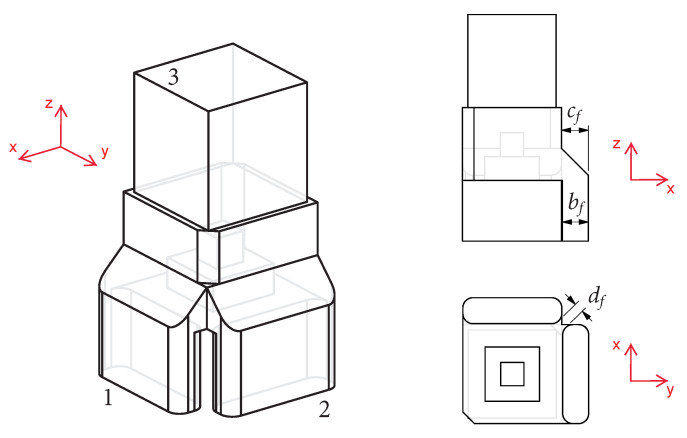
Isometric CAD view of the proposed very compact waveguide OMT with folded single-mode ports (**left**) and associated additional design parameters in side CAD (**top right**) and bottom CAD views (**bottom right**).

The design described in Section 2 is adapted here to include folded single-mode ports as proposed in [20]. Besides reducing the footprint of the component, this design variant has the advantage to be well adapted to additive manufacturing techniques, avoiding most overhanging parts in the original design when printing along the longitudinal direction of the feed assembly [16]. CAD views of the modified design are reported in Figure 5, introducing two additional design parameters: $b_{f}$, corresponding to the narrow wall dimension of the folded waveguides, and $c_{f}$, corresponding to the symmetric chamfer at the transition to the OMT. The optimized values for these parameters are $b_{f}=2.8$ mm and $c_{f}=2.9$ mm, which result in the folded waveguides being very close to each other. Implementing a mill radius of 1.1 mm, as also implemented in Section 2, the worst case distance between the waveguides is $d_{f}=1.05$ mm (see Figure 5), which is well in line with typical constraints of CNC milling and additive manufacturing techniques.

A full-wave model of this modified waveguide OMT was also implemented in Ansys HFSS, adding a milling radius of 1.1 mm to all outer edges, as carried out in the model of Section 2. Considering the good agreement between measured and simulated data demonstrated in the previous section, only numerical results are reported here. The change in the waveguide transition design slightly detunes the response of the component, specifically the XPD, with the resonance shifting to lower frequencies. A small adjustment of some design parameters is required. The updated design values for the folded case are reported in Table 2. The remaining parameters keep the same values as in Table 1.

**Figure 6:**
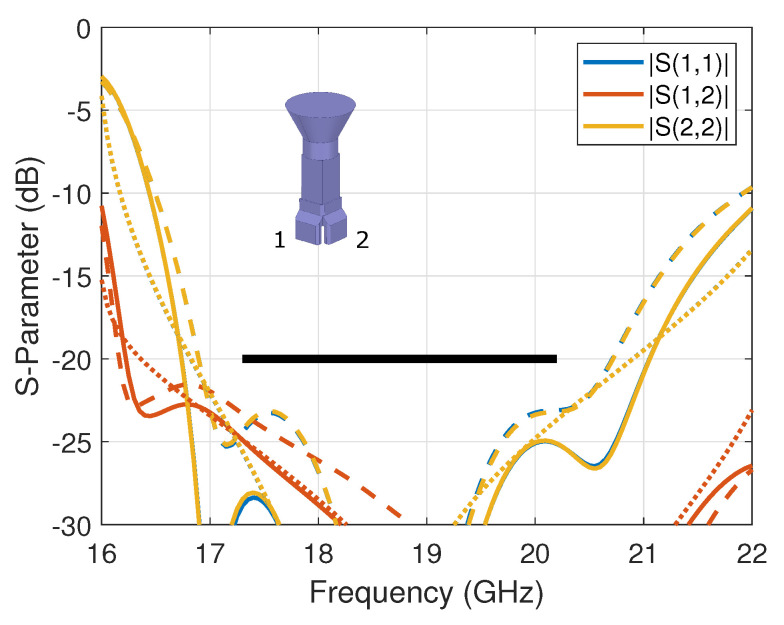
Simulated scattering parameters of the very compact waveguide OMT with folded single-mode ports assembled with a conical horn (*solid lines*) compared to the reference OMT design with straight single-mode ports (*dashed lines*). The simulated standalone OMT results are overlaid in *dotted lines*. The specifications are marked with a *black thick line*.

The simulated reflection and port-to-port coupling coefficients for this model are reported in Figure 6 in *solid lines*. For comparison, the simulation results of the reference case are also reported in *dashed lines*. Interestingly, this configuration was found to improve the scattering parameters, with worst case reflection and coupling coefficients of −24.9 dB and −24.8 dB, respectively, over the operating frequency band. For reference, the numerical results obtained for the standalone OMT with folded ports are also reported in Figure 6 in *dotted lines*, confirming the little impact of the horn antenna also in this case.

**Figure 7:**
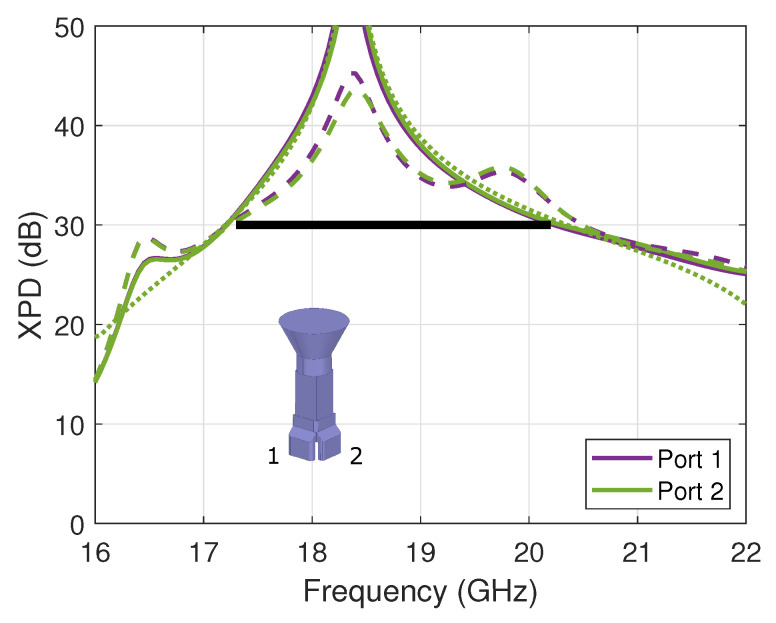
Simulated on-axis XPD of the very compact waveguide OMT with folded single-mode ports assembled with a conical horn (*solid lines*) compared to the reference OMT design with straight single-mode ports (*dashed lines*). The simulated standalone OMT results are overlaid in *dotted lines*. The specifications are marked with a *black thick line*.

The simulated on-axis XPD of the OMT assembled with the horn is reported in Figure 7 in *solid lines*. The results of the reference design are provided for comparison in *dashed lines*. The OMT design with folded ports achieved an XPD better than 30.1 dB over the nominal bandwidth, comparable to that of the reference OMT design. The performance of the standalone OMT with folded ports is reported in Figure 7 in *dotted lines*, confirming that this parameter is also mostly unaffected by the horn antenna. Note that this design was still constrained with the width of the probing section being set equal to the width of the standard WR42 waveguide and the common port being constrained by the available horn design, indicating that even better performances may be achieved by using these parameters as optimization variables. In particular, the component shows the potential to extend its operation bandwidth up to 21.2 GHz, corresponding to a fractional bandwidth of approximately 20% and covering also the portion of the spectrum in the K-band that is typically allocated for governmental applications.

A detailed comparison with alternative compact two-probe waveguide OMT designs is provided in Table 3, highlighting the benefits of the proposed solution. The dimension estimate in the xy-plane takes into account the space required to fold the waveguides along the longitudinal direction [20], i.e., the waveguide’s broad wall dimension in the case of side-coupling designs and the waveguide’s narrow wall dimension in the case of turnstile designs, as demonstrated in Section 3 for the proposed waveguide OMT design. Note that this dimension estimate is based on the waveguide cavities only and does not account for mechanical constraints, which are assumed to be the same for all designs. The specific waveguide OMT designs reported in this work were also constrained by interfaces required for tests. More compact designs are certainly achievable by implementing well-known techniques to reduce the waveguide cross-sections, such as ridges. The in-plane dimension is important for the design of closely spaced array antennas that are currently considered for broadband communication satellites, specifically in low and medium Earth orbit (LEO/MEO) systems with larger scanning ranges. Furthermore, the mechanical simplicity of the proposed OMT is essential for increasing the manufacturing yield in large arrays and for possibly adapting the design toward additive manufacturing techniques [12]. The waveguide OMT design with folded single-mode ports reported in Section 3 may be easily adapted for such purposes, as it presents very limited overhanging areas. There is also an interest in combining this improved waveguide OMT design with the four-way power divider described in [28] to further enhance its performance. Advantageously, the four-way power divider design does not require folding the single-mode ports, thus enabling further reduced array spacing in the order of 0.7$\lambda_{0}$, where $\lambda_{0}$ is the wavelength at the highest operating frequency.

## 4. Conclusions

A novel compact waveguide OMT was discussed. It adapts the concept of twofold rotationally symmetric OMTs recently introduced in the case of side-coupling OMT designs. A specific design was optimized for operations in the K-band, and the experimental results were in line with full-wave model predictions. It has also been demonstrated that folding the single-mode ports has limited impact on the overall performance of the waveguide OMT, providing a design more adapted to additive manufacturing techniques. Compared to equivalent designs with similar very small volumes, the proposed solution has advantages in presenting a much simpler mechanical design, which is expected to reduce its manufacturing cost and to improve its yield. This is particularly significant for the design of large closely spaced array antennas that are currently considered for LEO/MEO satellite communication applications. The proposed OMT may also find applications in the design of passive dual-polarized mechanically steered arrays used for the ground segment. Future works will address the design, manufacture, and test of a representative closely spaced array based on the proposed OMT solution.

## Figures and Tables

**Table 1:** Design parameters of the very compact two-probe waveguide OMT in the K-band with WR42 flanges.

Parameter	Value (mm)	Parameter	Value (mm)	Parameter	Value (mm)
$a_{\mathrm{WR}42}$	10.668	$d_{1}$	5.86	$z_{1}$	2.54
$b_{\mathrm{WR}42}$	4.318	$d_{2}$	2.50	$z_{2}$	2.60
*a*	9.50	$d_{3}$	3.18	$z_{3}$	3.45
*z*	4.40	*c*	1.25		

**Table 2:** Design parameters of the very compact waveguide OMT in the K-band with folded single-mode ports.

Parameter	Value (mm)	Parameter	Value (mm)	Parameter	Value (mm)
*z*	4.15	$d_{1}$	5.73	$z_{1}$	2.50
*c*	1.16	$d_{2}$	2.63	$z_{2}$	2.49
$b_{f}$	2.80	$c_{f}$	2.90	$d_{f}$	1.05

**Table 3:** Comparison with existing compact two-probe waveguide OMTs.

Reference	Type	Footprint (*xy*-Plane)	Length (*z*-Axis)	Manufacturing Complexity	Fractional Bandwidth	Return Loss	Port-to-Port Coupling	XPD
[4,5,6,7]	Side coupling	$0.7\lambda_{0}\times 1.5\lambda_{0}$	$>0.8\lambda_{0}$	Simple to moderate	20–30%	>24 dB	<−50 dB	>50 dB
[8,9,10,11]	Opposing ports	$0.7\lambda_{0}\times 1.8\lambda_{0}$	$∼0.8\lambda_{0}$	Moderate	20–30%	>20 dB	<−40 dB	Not reported
[17,18]	180${}^{\circ }$ septum	$0.7\lambda_{0}\times 0.7\lambda_{0}$	$∼2.5\lambda_{0}$	Simple to moderate	5–10%	>25 dB	<−35 dB	>35 dB
[19]	90${}^{\circ }$ septum and iris	$0.7\lambda_{0}\times 0.7\lambda_{0}$	$>3\lambda_{0}$	Simple to moderate	20%	>22 dB	<−22 dB	>30 dB
[21,22]	Two-probe transversal	$1.1\lambda_{0}\times 1.1\lambda_{0}$	$∼0.3\lambda_{0}$	High due to small features	10–15%	>20 dB	<−22 dB	>26 dB
**This work**	Two-probe transversal	$0.9\lambda_{0}\times 0.9\lambda_{0}$	$∼0.5\lambda_{0}$	Simple to moderate	15–20%	>19 dB	<−22 dB	>30 dB

Note: $\lambda_{0}$ is the wavelength at the highest operating frequency, as this is generally the limiting factor for phasedarray antenna design.

## Data Availability

Not applicable.
